# Synergistic Effect Evaluation and Mechanism Investigation of Vitamin B6 and B12 in Models of Neuroinflammation

**DOI:** 10.3390/ijms262210956

**Published:** 2025-11-12

**Authors:** Xixi Dou, Shiru Cai, Yingbo Liu, Junyan Wang, Huiying Li, Duo Gao

**Affiliations:** 1 Beijing Key Laboratory of Food Processing and Safety in Forestry, College of Biological Sciences and Technology, Beijing Forestry University, Beijing 100083, China; douxixi1545@bjfu.edu.cn (X.D.); qwer11632025@163.com (S.C.); y13051360209@163.com (Y.L.); 2 Hebei Province Key Laboratory of Sustainable Utilization and Development of Forest Food Resources, Beijing Forestry University, Xiong’an 071800, China; 3 College of Animal Science and Technology, Beijing University of Agriculture, Beijing 102206, China; 15064828900@163.com

**Keywords:** neuroinflammation, vestibular dysfunction, nanoencapsulation, synergistic combination, vitamin B6 and B12, PLP-1 protein

## Abstract

Neurological damage, a debilitating condition closely associated with chronic neuroinflammation, currently lacks disease-modifying treatments, with management limited to symptomatic relief. Vitamins B6 (VB6), B12 (VB12), and proteolipid protein 1 (PLP-1) exhibit multimodal neuroprotective and anti-inflammatory effects; however, their therapeutic potential is limited by low bioavailability and inadequate ability to cross the blood–brain barrier (BBB). To address these limitations, we developed an ursolic acid-based nanoparticle system for the intranasal co-delivery of VB6, VB12, and recombinant PLP-1. The PLP-1 model predicted by AlphaFold3 was used for molecular docking. The docking results confirmed high-affinity binding interactions with VB6 and VB12, elucidating the mechanistic basis of their synergy. In vitro studies using a glucose-deprived PC12 cell injury model identified an optimal synergistic molar ratio of 10:1:2 (VB6: VB12: PLP-1). This combination significantly upregulated neuroprotective markers (PLP-1 and PGC-1α) and downregulated the pro-inflammatory cytokine TNF-α. In a mouse model of neural damage, the nano-encapsulated combination therapy demonstrated improved pharmacokinetics and significantly attenuated neuroinflammation and oxidative stress in brain tissue. This was evidenced by lower TNF-α and IL-1β levels and elevated GSH and SOD concentrations compared to free drug controls. The treatment regimen showed no detectable hepatorenal toxicity. Our findings demonstrate that this nanoformulation represents a safe, effective, and promising disease-modifying strategy to treat vestibular dysfunction by synergistically targeting its underlying neuroimmunological mechanisms.

## 1. Introduction

Vestibular dysfunction encompasses various disorders, such as vestibular neuritis, benign paroxysmal positional vertigo, and Menière’s disease, all of which substantially impair balance and spatial orientation. The underlying mechanisms of these conditions are closely associated with chronic neuroinflammation in the central vestibular pathways, which is characterized by continuous microglial activation and increased release of pro-inflammatory cytokines (e.g., TNF-α and IL-1β), as well as activation of the pro-inflammatory transcription factor NF-κB. This inflammatory cascade causes neuronal damage and disrupts compensatory recovery processes [1,2]. Standard treatments, such as vestibular suppressants and physical rehabilitation, provide only symptomatic relief and do not target these underlying neuroinflammatory processes. Therefore, many patients experience chronic symptoms, highlighting the critical need for disease-modifying therapies that specifically target these neuroimmunological mechanisms [3,4].

Substantial preclinical evidence has identified vitamins B6 (2-Methyl-3-hydroxy-4,5-bis (hydroxymethyl) pyridoxine) and B12 (Methylcobalamin) as potent neuroprotective agents with multimodal anti-inflammatory properties [5,6,7]. In its active form, pyridoxal 5′-phosphate, VB6 serves as an essential coenzyme in neurotransmitter synthesis and immune regulation. Studies have indicated that it downregulates key inflammatory mediators, such as IL-1β, IL-6, and TNF-α, in macrophage-mediated models [7]. VB12, also known as cyanocobalamin in its common supplemental form and methylcobalamin in its biologically active forms, acts as a critical cofactor for methionine synthase and methylmalonyl-CoA mutase. Its deficiency increases homocysteine levels, which disrupt glial immune homeostasis and exacerbate neuroinflammation [8]. Ischemic stroke and epilepsy models have shown that VB6 and VB12 supplementation significantly attenuate neuroinflammation, reduce oxidative stress, and improve functional neurological outcomes [9,10]. Furthermore, myelin proteolipid protein 1 (PLP-1), a major structural component of myelin, is essential for axonal integrity and saltatory conduction [11,12]. It modulates neural resilience against inflammation, while its dysfunction is associated with higher vulnerability to neuroinflammatory damage [13].

In the field of neurological disease treatment, inefficient drug delivery and low bioavailability represent major bottlenecks to efficacy. Conventional drug delivery methods often face challenges such as poor drug solubility, short circulation time, difficulty in crossing the blood–brain barrier [14], and systemic side effects due to lack of specificity [15]. Nanoencapsulation technology offers a promising solution. This technique encapsulates active drugs within nanoscale carriers, significantly improving their physicochemical properties, enabling controlled release, prolonged efficacy, and enhanced targeting to diseased tissues through surface modification [16]. In particular, the use of natural active ingredients (e.g., ursolic acid) as nanocarrier materials not only enables efficient encapsulation and sustained release, but also further enhances drug efficacy through their inherent anti-inflammatory and antioxidant properties [17,18]. Therefore, ursolic acid-based nanoencapsulation systems offer promising prospects for the development of efficient and safe targeted therapies for neuroinflammation.

This study employed computer models to predict the molecular affinity of VB6 and VB12, followed by a multi-tiered experimental strategy to validate this synergistic combination. The anti-inflammatory and neuroprotective efficacy of different molar ratios was evaluated, after which a murine model of vestibular dysfunction was used for comprehensive in vivo studies. This included assessments of bioavailability, neuroinflammatory and oxidative stress responses, as well as systematic evaluations of hepatic and renal safety profiles. These analyses helped determine whether this concept represented a safe, effective treatment strategy.

## 2. Results

### 2.1. Investigation of the Affinity Between the PLP-1 and the Vitamins

**Figure 1:**
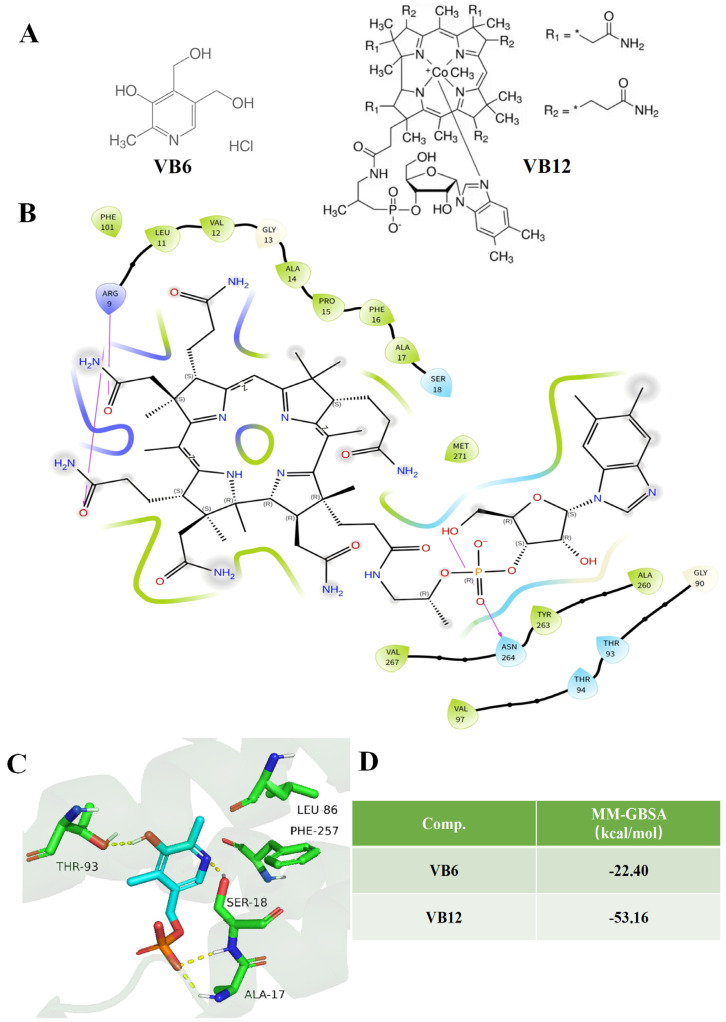
Predicted binding modes and affinities of VB6 and VB12 with PLP-1, as determined by molecular docking using the AlphaFold3-generated structure. (**A**) Chemical structures of VB6 and VB12. * stands for the binding point. (**B**) Combined mode analysis of VB12. (**C**) Combined mode analysis of VB6. (**D**) Affinity predictions for VB6 and VB12 binding to PLP-1 protein by Molecular Mechanics/Poisson-Boltzmann (Generalized Born) Surface Area (MM-GBSA).

As Figure 1 demonstrated, an AlphaFold3-predicted PLP-1 model was employed for molecular docking analysis, which revealed favorable binding affinities for both vitamins. VB12 bound in a larger hydrophobic pocket comprising ALA260, TYR263, and LEU11, among others, and established a hydrogen bond via its phosphate group with ASN264 (Figure 1B). Similarly, VB6 occupied a hydrophobic pocket created by the residues LEU86, PHE257, THR93, and ALA17, where it formed hydrogen bonds with SER18 and THR93 (Figure 1C). Using Molecular Mechanics/Poisson-Boltzmann (Generalized Born) Surface Area (MM-GBSA) method, the affinities of VB6, VB12 and PLP-1 protein were predicted as −22.40 kcal/mol (VB6/PLP-1) and −53.16 kcal/mol (VB12/PLP-1), indicating that both vitamins can bind PLP-1 directly. These results provided a structural basis for the subsequent design of a multicomponent therapeutic formulation that combined PLP-1 with VB6 and VB12 (Figure 1D). The docking region prediction results for small molecules of PLP-1 protein were demonstrated in Supplementary Figure S1 and the multiple protein model prediction results could be found in Supplementary Figure S2.

### 2.2. Determination of the Optimal VB6, VB12, and PLP-1 Combination Using Cell Experiments

**Figure 2:**
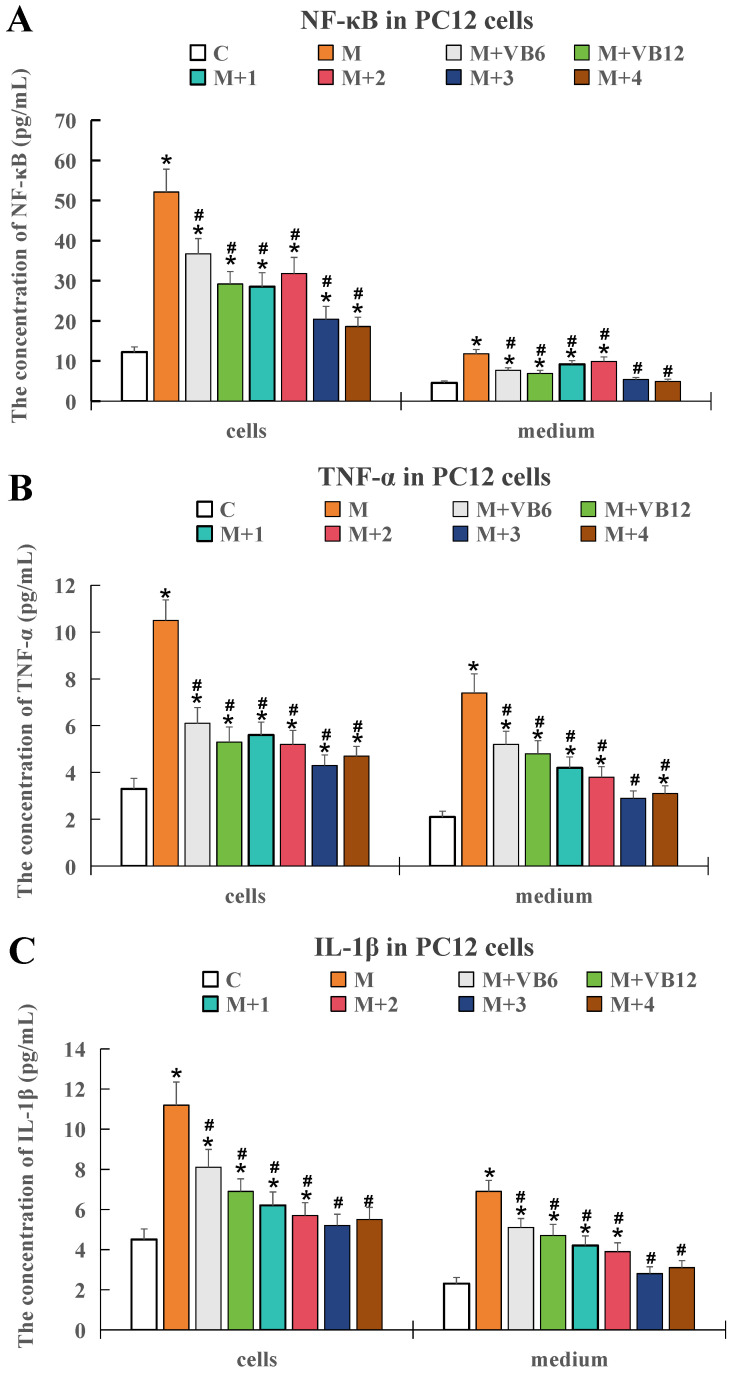
Expression levels of key inflammatory factors in PC12 cells under different treatment conditions. (**A**) Concentration of NF-κB in PC12 cells and medium. (**B**) Concentration of TNF-α in PC12 cells and medium. (**C**) Concentration of IL-1β in PC12 cells and medium. Data are presented as mean ± SD. * *p* < 0.05 compared to the control group; # *p* < 0.05 compared with the model group. n = 5. Specified molar ratios of the two vitamins VB6 and VB12 were represented as M + 1 (1:1), M + 2 (1:10), M + 3 (10:1), and M + 4 (20:1), respectively.

As shown in Figure 2, when compared with the control group, the expression levels of the three inflammatory factors in both cells and medium were significantly elevated in the model group (*p* < 0.05), indicating that the in vitro sugar and oxygen deprivation model successfully induced an inflammatory response. This confirmed the successful establishment of the cellular inflammation model and its suitability for subsequent therapeutic verification.

The results also showed that adding VB6 and VB12 at molar ratios of 1:10, 10:1, and 20:1 all effectively inhibited the upregulation of these inflammatory factors in the neuronal injury cell model (*p* < 0.05).

**Figure 3:**
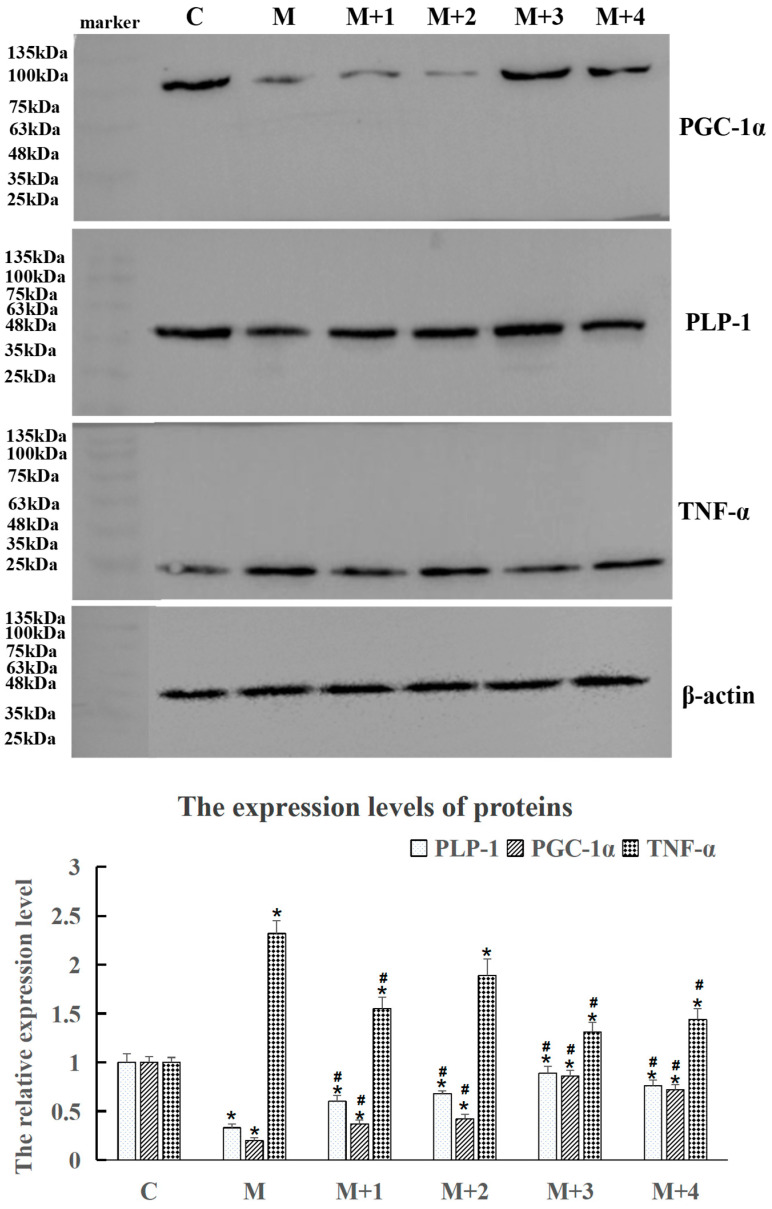
Western blot analysis showing the expression levels of PLP-1, PGC-1α, and TNF-α protein in the PC12 cells after treatment at different VB6 and VB12 molar ratios, with β-actin as an internal loading control. The relative protein expression levels were normalized to β-actin and are presented as mean ± SD. * *p* < 0.05 compared to the control group; # *p* < 0.05 compared with model group. n = 5. Specified molar ratios of the two vitamins VB6 and VB12 were represented as M + 1 (1:1), M + 2 (1:10), M + 3 (10:1), and M + 4 (20:1), respectively.

In Figure 3, the expression levels of PLP-1 and PGC-1α proteins in the model group significantly decreased (*p* < 0.05), while the level of TNF-α protein in the model group was significantly upregulated (*p* < 0.05), when compared with the control group. After treatment with VB6:VB12 combinations, the levels of PLP-1 and PGC-1α proteins were up-regulated, and the level of TNF-α was down-regulated compared with the model group (*p* < 0.05), illustrating that these combinations alleviate neurological damage through regulating the PLP-1/PGC-1α/TNF-α pathway. Interestingly, the VB6:VB12 combination at a 10:1 ratio was verified to most effectively modulate the protein expression in the injured neuronal cells, indicating that this optimal ratio has superior anti-inflammatory efficacy at the molecular level.

### 2.3. Validation of the Efficacy of the Intranasal Administration of Both the Free and Nano-Encapsulated Formulations

#### 2.3.1. ELISA Detection of the Inflammatory Factors and Oxidative Damage Factors in Mouse Brain Tissue

ELISA was employed to determine the inflammatory factors and oxidative damage markers in mice brain tissue with neurological impairment.

**Figure 4:**
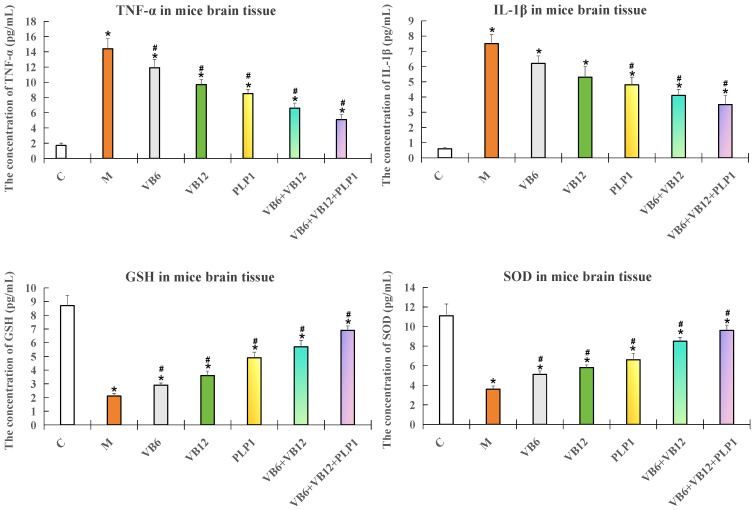
ELISA test results for four neuroinflammatory indicators (TNF-α, IL-1β, GSH, and SOD) in brain tissue of the modeled mice. Data are presented as mean ± SD. n = 5. * *p* < 0.05 compared to the control group; # *p* < 0.05 compared with model group.

As Figure 4 showed, the mice (n = 5 per group) were subjected to neuroinflammation and treated with VB6 only, VB12 only, PLP-1 only, the (VB6 + VB12) combination, or the (VB6 + VB12 + PLP-1) combination. In the model group, the levels of TNF-α and IL-1β significantly increased (*p* < 0.05), the levels of GSH and SOD significantly decreased (*p* < 0.05) compared with the control. No matter with the single treatments or combination treatments, the levels of TNF-α and IL-1β were downregulated and the levels of GSH and SOD were upregulated compared with the model group (*p* < 0.05), demonstrating the neuronprotective effects of vitamins and PLP-1 protein. The most effective group appeared to be the (VB6 + VB12 + PLP-1) group, indicating that PLP-1 enhanced the anti-inflammatory effect of VB6 and VB12.

**Figure 5:**
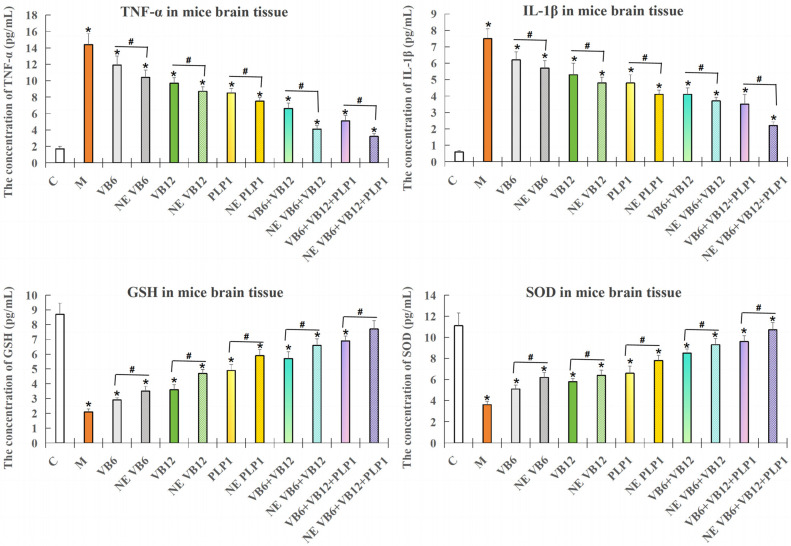
ELISA results for four neuroinflammation indicators (TNF-α, IL-1β, GSH and SOD) in brain tissue of the modeled PC12 mice. Data are presented as mean ± SD. * *p* < 0.05 compared to the control group; # *p* < 0.05 compared with the nanoencapsulation-free group. NE stood for nanoencapsulation treatment. n = 5.

In Figure 5, mice (n = 5 per group) were subjected to neurological injury and treated with both free and nano-encapsulated (NE) combinations. Results showed that the levels of TNF-α and IL-1β in NE-treated groups were lower than the ones in NE-free groups (*p* < 0.05), and the levels of GSH and SOD in NE-treated groups were higher than those in NE-free groups (*p* < 0.05), indicating that nanoencapsulation enhances the anti-inflammatory and antioxidative effects of the combination treatment in this model.

#### 2.3.2. Absence of Systemic Toxicity

**Figure 6:**
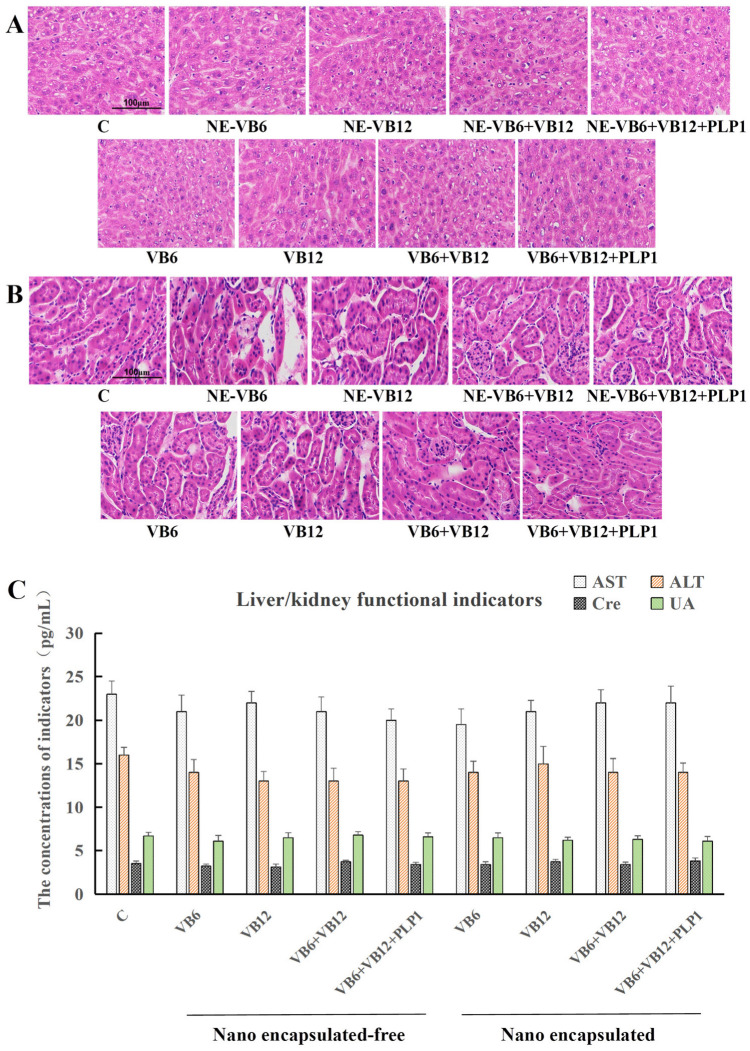
Biosafety assessment of different combinations of VB6, VB12, and PLP-1 protein. (**A**) HE staining of mice liver tissue. (**B**) HE staining of mice kidney tissue. (**C**) Detection of mice liver/kidney functional indicators (AST, ALT, Cre and UA). Data are presented as mean ± SD. NE stands for nanoencapsulation treatment. n = 5. The magnification was 200×.

Liver and kidney tissue staining showed no obvious pathological changes between the control group and the treatment groups, indicating that neither nanoencapsulation treatments nor nanoencapsulation-free treatments posed toxicity to liver and kidney tissues (Figure 6A,B). ELISA analysis of serum biochemical markers revealed no significant changes in the key hepatic (ALT and AST) and renal (Cre and UA) function indices across all treatment groups compared to the healthy control group (Figure 6C). These results confirmed the absence of detectable systemic hepatorenal toxicity after the administration of both free and nano-encapsulated experimental formulations, underscoring their excellent safety profile.

In Figure 6, no significant differences are evident between the control group and the serum levels of the liver (ALT and AST) and kidney function markers (Cre and UA) of the mice in the different treatment groups (*p* > 0.05), indicating no hepatorenal toxicity.

## 3. Discussion

As we know, Proteolipid Protein 1 (PLP1) is the most abundant protein in the central nervous system (CNS) myelin sheath and plays a crucial role in maintaining its structure, stability, and function [11,12]. It is a major structural component of myelin, and deficiency or dysregulation of PLP1 expression has been implicated in several neurodegenerative and demyelinating diseases, like hereditary spastic paraplegias, which always accompanying with neuroinflammation and immunodeficiencies [13]. Thus, the methods to ameliorate inflammation and enhance immunity by up-regulating PLP1 expression might play roles in treating neurodegenerative and demyelinating diseases. In the present study, we revealed the effects of the VB6 + VB12 + PLP-1 combination in a model of vestibular dysfunction (a form of neurological damage), indicating that this combination exerted inhibitory effects on inflammatory signaling. Previous studies have confirmed that the active form of VB6 and PLP1 can markedly suppress NLRP3 inflammasome activation, leading to decreased IL-1β production and attenuated responsiveness to LPS/ATP stimulation [19]. Similarly, VB6 has been shown to inhibit NF-κB nuclear translocation and IκBα degradation in LPS-stimulated RAW 264.7 macrophages, leading to reduced expression of pro-inflammatory genes such as *iNOS* and *COX-2* [20]. Our results align with these previous findings, suggesting that VB6 functions primarily by suppressing NF-κB and NLRP3 pathways.

Recent studies have revealed that VB12 plays a critical role in microglial regulation. An iScience report showed that VB12 can reprogram microglial gene expression by modulating fatty acid metabolism and mitochondrial function, which alleviates neuroinflammation and improves outcomes in ischemic stroke models [9]. An animal model of VB12 expression deficiency displays impaired motor performance, reduced neuronal survival, altered choline metabolism, and elevated apoptosis [21]. This finding underscores the essential role of VB12 in neuronal survival and metabolic homeostasis [21]. Taken together, these data indicate that VB12 likely contributes to neuroprotection in our system by promoting an anti-inflammatory and metabolically favorable microglial phenotype.

Our molecular docking results revealed that VB6 and VB12 could occupy distinct hydrophobic pockets of PLP-1 and form stabilizing hydrogen bonds, suggesting that these vitamins might influence PLP-1 conformation and function. Given that altered PLP-1 activity had been linked to axonal integrity and neural resilience against inflammation [11,12,13], the observed binding interactions of vitamins and PLP-1 protein might provide a structural basis for the immunomodulatory effects observed in this study.

Our findings revealed upregulation of PGC-1α, suggesting that mitochondrial and antioxidant pathways were involved in the observed protective effects. This result was consistent with the report showing that VB6 could mitigate cadmium-induced oxidative stress and memory deficits via activation of the p-JNK/Nrf2/NF-κB pathway [22]. Conversely, VB6 deficiency leads to reduced PLP-1 levels in the brain, elevated oxidative stress, lipid peroxidation, apoptosis, and increased markers of neurodegeneration [23]. These results reinforce the notion that VB6 and VB12 not only attenuated inflammation but also enhanced mitochondrial function and antioxidant defenses, thereby providing a dual mechanism of neuroprotection.

Nasal administration provides a recognized non-invasive route to bypass the blood–brain barrier. Multiple studies have highlighted that nanoparticle-based carriers, including lipid nanoparticles, nanoemulsions, and nanostructured lipid carriers, can significantly enhance brain exposure, prolong mucosal retention, and improve tissue targeting [24,25,26]. In our study, the nano-encapsulated formulation exhibited stronger effects than the free drug in reducing brain inflammation and enhancing antioxidant markers, consistent with reports that nanoparticles improve nasal mucosal penetration and increase transport efficiency through the olfactory and trigeminal pathways [24,25,26].

Encapsulation offers several advantages: (i) protection of labile molecules from enzymatic degradation and enabling controlled release, thereby prolonging bioavailability [24,26]; (ii) improved mucoadhesion and cellular uptake, especially when nanoparticles are surface-modified with PEG or ligands to enhance epithelial interaction [25,27]; (iii) enhanced accumulation in target brain regions, as demonstrated in nanoparticle-based delivery of ursolic acid to glioblastoma and subarachnoid hemorrhage models [18,28]. Together, these mechanisms plausibly explain why the nano-formulation exhibited superior therapeutic outcomes in our animal model.

The superiority of the nano-formulation led to testable hypotheses: that Nanoencapsulation extended nasal mucosal retention, enhances exposure of vestibular-related nuclei via olfactory/trigeminal pathways, and reduced systemic clearance while increasing CNS area under the curve (AUC). These predictions could be validated by LC-MS quantification of drug distribution in nasal mucosa, plasma, and brain tissues, combined with imaging studies using fluorescent or radiolabeled nanoparticles [24,27].

To sum up, vitamins B6 and B12 could up-regulate the expression level of PLP1 protein and then suppress the neuroinflammation in the present study, while the particular mechanisms were still not elucidated. For PLP1 deficiency-related disorders, the wmN1 enhancer is proved to be a critical therapeutic target, Hamdan find that it is essential for high-level human PLP1 expression via the native promoter [29], while Patyal confirms its indispensability for mouse PLP1 expression in the central, peripheral, and enteric nervous system, interventions like correcting wmN1 defects or activating its transcription can restore physiological PLP1 levels [30,31]. Therefore, whether vitamins B6 and B12 increase PLP1 expression level through regulating the wmN1 enhancer deserve the further validation. Referring to another key point, whether these vitamins are essential for remyelination, Miller shows that VB12 is essential as a co-factor for myelin synthesis and has immunomodulatory and neurotrophic effects, suggesting that adequate levels are vital for creating a favorable environment for remyelination, but not through direct upregulation of PLP1 transcription [32].

Additionally, several limitations should be acknowledged. Firstly, the predicted binding interactions between VB6/VB12 and PLP-1 remained computational and required further validation through experimental methods such as SPR, ITC, or mutagenesis. Secondly, the small sample size in the animal study limited statistical power, and functional behavioral outcomes such as balance assessment should be included in future studies. Thirdly, while data support improved efficacy with Nanoencapsulation, direct pharmacokinetic and biodistribution studies are needed to fully confirm the advantages of the delivery system [24,26].

## 4. Materials and Methods

### 4.1. Determination of the Optimal Combination of VB6, VB12, and PLP-1 in the Drug Using Cell Experiments

#### 4.1.1. Cell Culture

The neuronal model in this study consisted of pheochromocytoma 12 (PC12 cells, rat adrenal pheochromocytoma), which were purchased from ATCC (Manassas, Virginia, USA) and maintained in culture medium comprising RPMI 1640 supplemented with 10% fetal bovine serum (FBS), 5% horse serum (HS), and 1% penicillin/streptomycin. All cultures were incubated in a humidified atmosphere at 37 °C and 5% CO_2_. The growth medium was refreshed daily, and the cells were subcultured every 2 days. The above reagents were purchased from Solarbio Company (Beijing, China).

#### 4.1.2. Construction of the Neurons Injury Model

The in vitro neuronal injury model was established by replacing the normal culture medium with a sugar-free medium (2 mL per well in a 6-well plate). Next, the cells were placed in a sealed anaerobic glass container for 2 h to simulate ischemic conditions, after which the medium was switched back to the normal culture medium. At the time of medium replacement, VB6, VB12, or a combination of the two vitamins at specified molar ratios of 1:1 (M + 1), 1:10 (M + 2), 10:1 (M + 3), and 20:1 (M + 4) was added to the respective wells, M stood for injury model treatment, and the above ratios stood for the molar concentrations of two vitamins (VB6/VB12), 1:1 group stood for 1 μM VB6:1 μM VB12, 1:10 group stood for 1 μM VB6:10 μM VB12, 10:1 group stood for 10 μM VB6:1 μM VB12, 20:1 group stood for 20 μM VB6:1 μM VB12. The cell samples were collected after 24 h of vitamin treatment to detect the inflammatory factor expression levels (Figure 3) and determine the optimal dose combination.

#### 4.1.3. Western Blot Analysis

After treatment, the cells were collected and lysed using lysis buffer. For PLP-1 protein extraction, the Thermo Scientific NE-PER membrane protein separation kit was used as follows. Firstly, cell samples were washed twice with pre-cooled PBS to remove residual culture medium, pre-cooled lysis buffer (containing protease inhibitor) was added into the samples and lysed on ice for 20 min, secondly, the samples were centrifuged at 12,000× *g* for 10 min to collect the supernatant (total protein), we used high-speed centrifugal columns or specific resin to adsorb membrane proteins, high-purity target proteins were obtained through washing and elution steps, thirdly, the samples were centrifuged at 12,000× *g* for 7 min to collect the bound membrane proteins, fourthly, after quantification using BCA method, the membrane proteins samples were stored at −20 °C to avoid repeated freeze–thaw cycles. For other proteins, the total proteins were gathered after cells lysis. Next, 300 μL of 5× loading buffer was added to each lysate sample, which was then denatured for 15 min. The protein expression levels of the PLP-1, PGC-1α, and TNF-α were quantified via Western blot analysis, using β-actin as the loading control.

The electrophoresis tank was filled with 1× Tris-glycine electrophoresis buffer, after which the samples and pre-stained rainbow protein marker were loaded onto the gel. Electrophoresis was initiated at 120 V for 15 min, followed by a continued reaction at the same voltage for approximately 40 min until the protein markers were completely separated.

A PVDF membrane was activated via soaking in anhydrous methanol for 1 min, after which it was immersed in 1× transfer buffer along with sponges and filter paper. The order of the transfer stack assembly included sponge, filter paper, gel, membrane, filter paper, and sponge, from cathode to anode. After electrophoretic transfer at 110 V for 1.5 h, the membrane was briefly rinsed and blocked with 10% skim milk for 1 h. It was then washed three times with 1× TBST for 5 min each, followed by incubation with the primary antibody for 2 h. After another series of three TBST washes, the membrane was incubated with goat anti-rabbit IgG-HRP in 1× TBST for 1 h. After three final washes with TBST, the membrane was treated in the dark with 1 mL of ECL developing reagent. The chemiluminescent signals were captured using a dedicated imaging system, and the relative protein expression levels were normalized to β-actin.

### 4.2. Affinity Detection Between the PLP-1 Protein and Vitamins (B6/B12)

#### 4.2.1. PLP-1 Protein

The recombinant PLP-1 protein was prepared by Beijing Tsingke Biotech Co., Ltd. (Beijing, China), according to the publicly available protocol detailed in the article “Human myelin proteolipid protein structure and lipid bilayer stacking” [33].

#### 4.2.2. Molecular Docking Analysis of the PLP-1-Vitamin Interactions

The amino acid sequence of the PLP-1 protein from Uniprot (https://www.uniprot.org/) was downloaded and submitted to the AlphaFold3 server (https://golgi.sandbox.google.com/), five interaction models were predicted and the Model 0 with the highest ranking score for molecular docking was selected, the model we employed for molecular docking was Human Complement Component C3c, and the corresponding PDB ID was 2A74. In detail, during protein preparation, the hydrogen bond network was optimized, and the energy of the protein system was minimized. Small molecule preparation involved generating three-dimensional structures, minimizing energy, and creating multiple conformations to improve the probability and precision of successful docking. Then, the SiteMap module of Maestro was utilized to predict binding pockets for the small molecules using default parameters, followed by the generation of receptor grid files. The most probable binding pocket was identified based on the predicted pocket location, size, and small molecule docking scores, while other parameters were maintained at their default values. For the molecular docking phase, the prepared grid files and small molecule ligands were subjected to docking using the ligand docking module of the software. The optimal binding mode was determined by analyzing the docking scores and the structural rationality of the small molecule binding conformations. Finally, the figures using the open-source version of PyMOL software (2.6) were generated.

Docking parameters of VB6 were:

center_x = −5.6
center_y = −11.3
center_z = 9.1
size_x = 15.8
size_y = 18.4
size_z = 15.2

Docking parameters of VB12 were:

center_x = −7.9
center_y = −22.8
center_z = 11.3
size_x = 26.7
size_y = 30.8
size_z = 22.6

### 4.3. In Vivo Efficacy Validation

#### 4.3.1. Animal Grouping and Drug Administration

Six-week-old male ICR mice were selected and randomly divided into 10 experimental groups (n = 5 per group) (Table 1). The groups included control, model, and various treatment groups that received free or nano-encapsulated drugs via intranasal administration every day, the total treatment time was 5 weeks (35 days), and the dosage of each treatment group was demonstrated in Table 1.

All active treatments were administered intranasally before model establishment. The optimal molar ratio (VB6:VB12:PLP-1 = 10:1:2) was maintained across the combination therapy groups (Groups 5, 6, 9, and 10).

#### 4.3.2. Vestibular Injury Model Establishment

After general anesthesia of isoflurane (2.5%, inhalation in the isolation box), the mice received 0.03 mL of a 100 mg/mL arsanilic acid solution dissolved in warm water at 37 °C via intratympanic injection. The mice were positioned with the injected ear upward for 5 min, after which sterile cotton wool was used to seal the injection site. After recovery from anesthesia, the mice were secured on a rotating rod by their tails, positioned head-down, and laterally rotated for approximately 90 s, with one rotation every 2 s. To confirm successful model establishment, the mice were transferred to a clean cage and monitored for behavioral indicators such as dizziness, nausea, vomiting, nystagmus, nystagmus-like head swaying, head-tilting toward the injected side, unsteady gait, and a tendency to fall or lean toward the affected side.

After the experimental procedures, blood samples were collected via retro-orbital bleeding, and the mice were euthanized by cervical dislocation. The liver, kidneys, brain, and other relevant tissues were harvested and preserved for subsequent analysis.

The animal experiments in this study were approved by the Ethics Committee of the Sinoresearch Biotechnology Co., Ltd. (Beijing, China; protocol: ZYZC20240720S; approval date: 20 July 2024), and was performed following standard guidelines. This work did not involve the use of material from human subjects.

#### 4.3.3. Preparation of the Nanoparticles

An ultrasonic emulsification method was employed to prepare the ursolic acid nanoparticles as follows, firstly, a 10 mg/mL solution of ursolic acid (solvent: methanol) was prepared, 2 mg of vitamins was dissolved in 1mL of the aforementioned ursolic acid methanol solution to obtain a “ursolic acid isocoumarin” mixture; secondly, two concentrations of polyvinyl alcohol (PVA) solution were prepared, 2.5% PVA solution (for pre emulsification) and 0.3% PVA solution (for subsequent dispersion stabilization), under vortex stirring conditions, the mixture of “ursolic acid/vitamins” was added into 2.5 mL of 2.5% PVA solution, and mixed evenly to form a pre emulsion; thirdly, ultrasonic emulsification treatment was performed on the pre emulsion with an ultrasonic time of 90 s, and the mixture was quickly transferred to 35 mL of 0.3% PVA solution and stirred continuously for 12 h on a magnetic stirrer to promote stable formation of nanoparticles; fourthly, the above mixture was transferred to a centrifuge tube and centrifuged at 4 °C and 12,000 r/min for 30 min, then the precipitate was gathered as the ursolic acid nanoparticles embedded with vitamins. The morphology of the prepared nanoparticles was examined via scanning electron microscopy (SEM), which confirmed successful formation. The encapsulation efficiency of VB6 and VB12 was determined using an Agilent 1290 high-performance liquid chromatography (HPLC) system (Agilent Technologies, Santa Clara, CA, USA). The detailed HPLC parameters are summarized in Table 2.

#### 4.3.4. Preparation of the Intranasal Formulations

All intranasal formulations were prepared in sterile conditions. Table 3 summarized the final concentrations of the active compounds in the vehicle (physiological saline).

In Table 3, the formulations were designed to maintain the optimal synergistic molar ratio (VB6:VB12:PLP-1 = 10:1:2) identified via in vitro studies.

### 4.4. Post-Treatment Analysis

Blood, liver, kidney, and brain tissue samples were collected from the mice and analyzed as described in the subsequent sections.

#### 4.4.1. Detection of the Vitamin Concentrations in the Mouse Blood

A 100 μL aliquot of mouse plasma was pipetted into a 1.5 mL centrifuge tube, followed by the addition of 400 μL of methanol for protein precipitation and extraction. The tubes were sealed tightly, after which the mixture was sonicated for 10 min and centrifuged at 12,000 rpm and 10 °C for 10 min. Next, 300 μL of the subsequent supernatant in each sample was carefully transferred to a new tube.

#### 4.4.2. ELISA Detection of the Inflammatory and Oxidative Stress Factors in the Mouse Brain Tissue

Commercial mouse-specific ELISA kits were employed to quantify the inflammatory and oxidative stress factors. A plate was incubated overnight at 4 °C in PBS with 1–5 μg/mL of the capture antibody. The plate was washed and blocked for 1 h at 37 °C by adding 200 μL of blocking buffer to each well. Serially diluted standards and appropriately diluted plasma samples (e.g., 1:2) were added in duplicate (100 μL/well) and incubated for 2 h at 37 °C. The plate was washed, followed by the addition of a biotin-conjugated detection antibody and incubation for 1 h at 37 °C. The plate was washed again, after which HRP-streptavidin was added and incubated in the dark for 30 min at 37 °C. The color was developed in the dark for 15 min at RT using TMB substrate (100 μL/well). The reaction was terminated with stop solution (50 μL/well), and the absorbance was immediately measured at 450 nm. The sample concentrations were determined by interpolating the mean absorbance of the duplicates from the standard curve.

#### 4.4.3. ELISA Detection of the Liver and Kidney Function Markers in the Mouse Serum

The experimental procedure for serum marker analysis was consistent with the protocol detailed above for the ELISA detection of the inflammatory and oxidative stress factors in the mouse brain tissue.

### 4.5. Hematoxylin and Eosin (HE) Staining of Liver/Kidney Tissues

A HE staining kit was used to perform pathological staining of liver, kidney, and intestinal tissues. The wax of the tissue slices was removed, the slices were hydrated, and the cell nuclei were stained with hematoxylin and eosin for 20 min. Then the slices were washed with water and differentiated with hydrochloric acid ethanol, and the cytoplasm was stained with eosin to make it red. Finally, the slices were dehydrated, made transparent, and sealed. The morphology and structure of the tissue cells were clearly observed under a microscope.

### 4.6. Statistical Analysis

The data were presented as the mean ± standard deviation (SD). The sample size “n” represented the number of independent biological replicates (mice) per treatment group. One-way analysis of variance (ANOVA) followed by Tukey’s post hoc test was used for comparisons across ten treatment groups. The thresholds for statistical significance were defined as follows: * *p* < 0.05. The GraphPad Prism software (version 9.3) was used for graphing and statistical analyses.

## 5. Conclusions

This study successfully designed and validated a novel combinatorial nanotherapy targeting neuroinflammation in vestibular dysfunction. The results showed that combined treatment with VB6, VB12, and PLP-1 protein (molar ratio of 10:1:2) via an ursolic acid-based nanoemulsion was a highly effective and safe therapeutic strategy, demonstrating strong anti-inflammatory and antioxidant effects, with no observable liver/kidney toxicity. This approach exhibits considerable promise for the clinical treatment of neuroinflammatory conditions. Future efforts will focus on clarifying the precise immunomodulatory pathways involved and advancing this therapeutic strategy toward clinical implementation.

## Figures and Tables

**Table 1:** Design of experimental groups and dosing amounts.

Group	Treatment Description	Formulation	Dosage
1	Control	-	No treatment
2	Model	-	Model establishment only
3	VB6	Free drug	VB6 (2 μmol/kg body weight (b.w.))
4	VB12	Free drug	VB12 (0.2 μmol/kg b.w.)
5	VB6 + VB12	Free drug	VB6 (2 μmol/kg b.w.) + VB12 (0.2 μmol/kg b.w.)
6	PLP-1 + VB6 + VB12	Free drug	PLP-1 (1 μmol/kg b.w.) + VB6 (2 μmol/kg b.w.) + VB12 (0.2 μmol/kg b.w.)
7	Nano-VB6	Nano-encapsulated	VB6 (2 μmol/kg b.w.)
8	Nano-VB12	Nano-encapsulated	VB12 (0.2 μmol/kg b.w.)
9	Nano-VB6-VB12	Nano-encapsulated	VB6 (2 μmol/kg b.w.) + VB12 (0.2 μmol/kg b.w.)
10	Nano-PLP-1-VB6-VB12	Nano-encapsulated	PLP-1 (1 μmol/kg b.w.) + VB6 (2 μmol/kg b.w.) + VB12 (0.2 μmol/kg b.w.)

**Table 2:** The HPLC parameters and conditions employed for the detection of vitamin encapsulation.

Parameter	Condition	
Column	Agilent ZORBAX SB-C18 (4.6 × 150 mm, 5 μm) (Agilent Technologies, Santa Clara, CA, USA)	
Mobile phase	A: 0.2% formic acid in water B: Acetonitrile	
Flow rate	0.2 mL/min	
Gradient elution	Time (min)	%B
	0–0.5	30
	0.5–2	30 → 80
	2–3	80 → 98
	3–5	98
	5–7	98 → 30
	7–9	30
Run time	9 min	
Injection volume	2 μL	
Column Temperature	35 °C	
Detection Wavelength	330 nm	

**Table 3:** Composition of the intranasal formulations including VB6, VB12, PLP-1 protein, and vehicle.

Component	Concentration	Note
Vitamin B6 (VB6)	25 µM	Molar ratio of VB6:VB12 = 10:1
Vitamin B12 (VB12)	2.5 µM	
PLP-1 protein	5 µM	Molar ratio of VB6:PLP-1 = 5:1
Vehicle	Physiological saline	-

## Data Availability

The original contributions presented in this study are included in the article/Supplementary Materials. Further inquiries can be directed to the corresponding authors.

## Supplementary Materials

The following supporting information can be downloaded at: https://www.mdpi.com/article/10.3390/ijms262210956/s1.

## Abbreviations

The following abbreviations are used in this manuscript:

ALA	Alanine
ALT	Alanine Aminotransferase
ANOVA	Analysis of Variance
ASN	Asparagine
AST	Aspartate Aminotransferase
ATP	Adenosine Triphosphate
AUC	Area Under the Curve
B6/VB6	Vitamin B6
B12/VB12	Vitamin B12
BBB	Blood–Brain Barrier
CHO	Chinese Hamster Ovary
CNS	Central Nervous System
CoA	Coenzyme A
COX-2	Cyclooxygenase-2
Cre	Creatinine
ECL	Enhanced Chemiluminescence
ELISA	Enzyme-Linked Immunosorbent Assay
FBS	Fetal Bovine Serum
GSH	Glutathione
HRP	Horseradish Peroxidase
HS	Horse Serum
ICR	Institute of Cancer Research
IκBα	Inhibitor of Kappa B Alpha
IgG	Immunoglobulin G
IL-1β	Interleukin-1 beta
IL-6	Interleukin-6
iNOS	Inducible Nitric Oxide Synthase
ITC	Isothermal Titration Calorimetry
LC-MS	Liquid Chromatography-Mass Spectrometry
LEU	Leucine
LPS	Lipopolysaccharide
NF-κB	Nuclear Factor Kappa-Light-Chain-Enhancer of Activated B cells
NLRP3	NOD-Like Receptor Family Pyrin Domain Containing 3
Nrf-2	Nuclear Factor Erythroid 2-related Factor 2
P	Probability Value
PBS	Phosphate-Buffered Saline
PC12	Pheochromocytoma 12
PEG	Polyethylene Glycol
PGC-1α	Peroxisome Proliferator-Activated Receptor Gamma Coactivator 1-alpha
PHE	Phenylalanine
PLP-1	Proteolipid Protein 1
p-JNK	Phospho-c-Jun N-terminal Kinase
PVDF	Polyvinylidene Fluoride
Raw 264.7	Raw 264.7 Cell Line
RPMI 1640	Roswell Park Memorial Institute 1640
SD	Standard Deviation
SEM	Scanning Electron Microscopy
SER	Serine
SOD	Superoxide Dismutase
SPR	Surface Plasmon Resonance
TBST	Tris-Buffered Saline with Tween-20
THR	Threonine
TMB	3,3′,5,5′-Tetramethylbenzidine
TNF-α	Tumor Necrosis Factor-alpha
TYR	Tyrosine
UA	Uric Acid
V	Volt
HPLC	High-Performance Liquid Chromatography
