# Supplementary material for: Synergistic Effect Evaluation and Mechanism Investigation of Vitamin B6 and B12 in Models of Neuroinflammation
Source: Int J Mol Sci. 2025 Nov 12;26(22):10956. doi: 10.3390/ijms262210956 (PMC12652146; doi:10.3390/ijms262210956)
Supplement: Supplementary file 1 [file ijms-26-10956-s001.zip › ijms-3962410-supplementary.pdf]

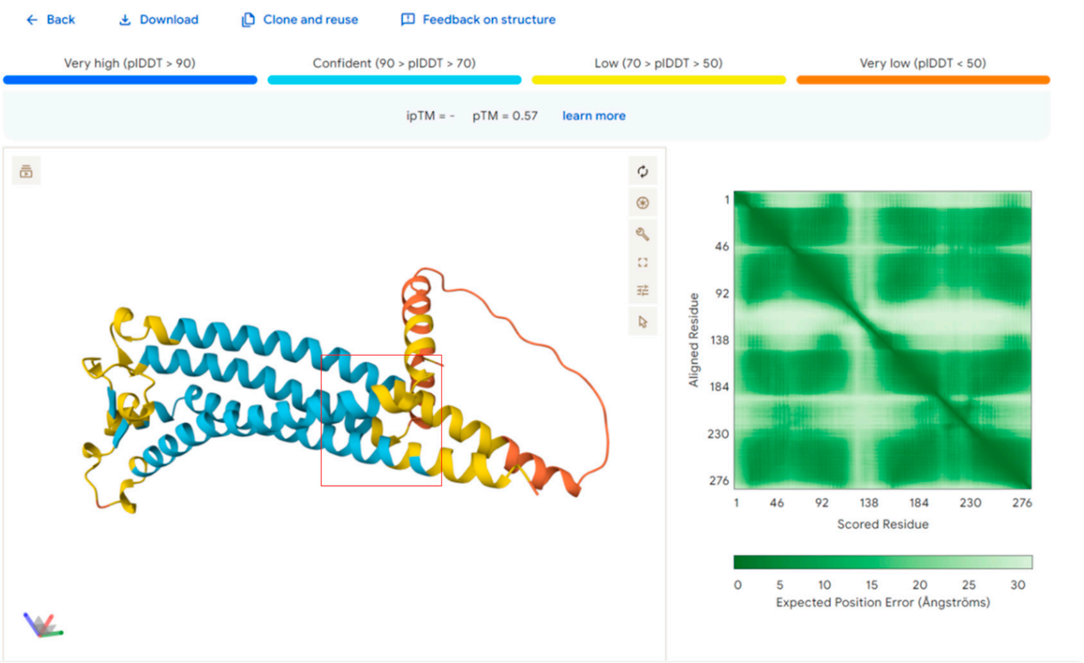

Supplementary Figure S1. The docking region prediction for small molecules of PLP-1 protein.

The docking region for small molecules was approximately in red box, currently at a binding affinity range of 90-70 and 70-50. Results demonstrated that Core domain pLDDT>80 was trustworthy, and flexible ring 50-70 required MD correction, PTM 0.57 indicated that the overall folding and the overall topology were correct. Referring to PAE matrix (bottom right corner), the diagonal area appeared to be dark blue, and the relative positions between residues were reliable.

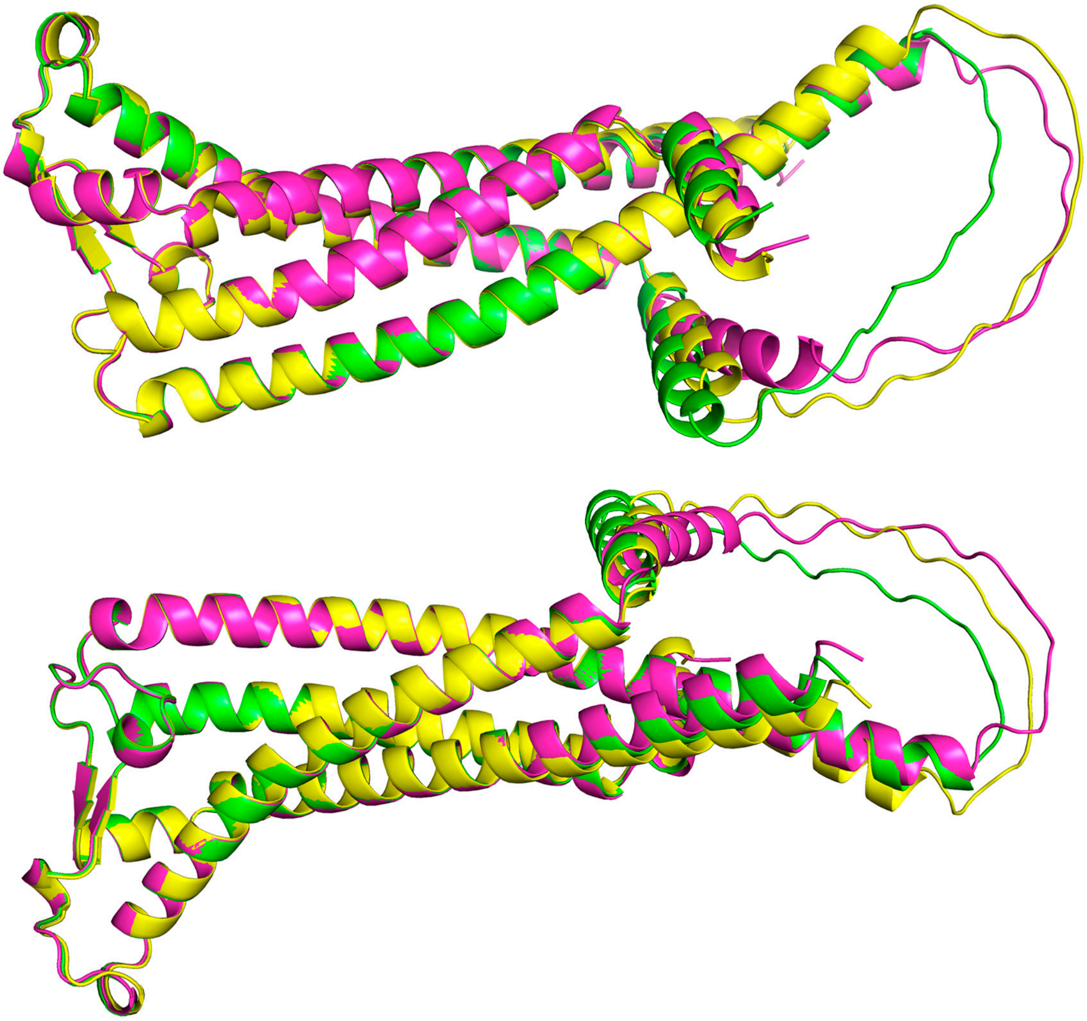

Supplementary Figure S2. Multiple protein model prediction results.

The docking given that the protein does not have a publicly available crystal structure, we had overlaid multiple protein model predictions and found that the results were generally consistent and stable.
